# Construction and application of standardized training effect evaluation system for new nurses in operating room

**DOI:** 10.1002/hcs2.75

**Published:** 2023-12-10

**Authors:** Xiaoli Liu, Yanshu Wei, Jin Pei, Xiaozhou Wu

**Affiliations:** ^1^ Operating room of Peking University People's Hospital Beijing China

**Keywords:** operating room nurse, new nurses, standardized training, evaluation system, Kirkpatrick model

## Abstract

**Background:**

This study aims to develop and validate a Structured Training Effectiveness Evaluation (STEE) tool based on the Kirkpatrick model for newly graduated registered nurses in the operating room in China.

**Methods:**

The first phase will involve focus group and individual interviews with nursing educators and newly graduated registered nurses selected using purposive sampling. The data will be analyzed thematically to identify key components necessary to develop the STEE tool. The second phase will develop and validate the STEE tool through a panel of experts using the Delphi method. The item weights will be determined with the analytic hierarchy process technique. The third phase will involve implementation and evaluation of the STEE tool with an exploratory, nonexperimental, and comparative analysis. Descriptive and inferential statistical analyses will be performed with SPSS version 23.

**Results:**

The STEE tool for newly graduated registered nurses in the operating room will be useful for evaluating training effectiveness during standardized training. The results obtained with this tool will clarify the effectiveness of training, thereby helping transform nursing students into competent nurses.

**Conclusion:**

In this way, this study will provide practical guidance for improving standardized training programs and help newly graduated nurses manage their transition to the clinical work environment and remain in their posts.

## Graphical abstract

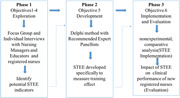

**Abbreviations**
AHP: analytic hierarchy process
STEE: Structured Training Effectiveness Evaluation

## 1 INTRODUCTION

A considerable number of new nurses are moving into operating room nursing posts as the turnover of nursing staff increases. Newly graduated registered nurses (i.e., nurses with <2 years of work experience, including new graduates and graduates with <2 years of experience in other hospitals) have certain deficiencies compared with senior nurses, such as weak specialized nursing skills, poor work planning, and low risk awareness. Therefore, training for new nurses is critical to ensure the quality of clinical nursing. Moreover, the importance of training for new nurses is emphasized in the Training Outline for New Nurses (Trial) [1] issued by the National Health and Family Planning Commission in February 2016. Specifically, this document requires all hospitals to provide standardized training for new nurses. However, the medical environment and working systems are not exactly the same across hospitals, and their training methods and content may differ. Nevertheless, training for new nurses in all hospitals should achieve the same goals; that is, standardized training in clinical nursing work, basic theories, knowledge, and skills, professional nursing ethical qualities, communication ability, emergency handling capacity, and the ability to independently provide standard quality nursing services for patients.

Evaluation of effectiveness is a critical link in training that offers a yardstick to measure whether training has achieved the expected goals and provides guidance for organizers, participants, and beneficiaries of training projects. The Quality Management‐Training Guide (IS010015) issued by the International Standardization Organization in 1999 stated that a training evaluation mechanism should be established to address the lack of training effectiveness evaluation [2]. However, methods of evaluating training effectiveness and continually improving training remain in the exploratory stage. China has not yet developed a systematic and operable training effectiveness evaluation system, thereby failing to guarantee the comparability and popularization of training effectiveness. To some extent, this hinders the scientific process of human resources management for nursing. Therefore, this study aims to develop an evaluation system that meets the needs of medical models and clinical posts in China. The system will be based on the Kirkpatrick model and use mixed methods to evaluate new operating room nurses at different stages of standardized training. In this way, this study will provide practical guidance for improving standardized training programs and help newly graduated nurses manage their transition to the clinical work environment and remain in their posts.

### 1.1 Domestic evaluation index of standardized training for operating room nurses

Standardized training for operating room nurses has generated extensive attention among researchers because of its uniqueness. Many researchers have focused on developing reasonable training programs. However, most training evaluation methods were based on the hospital training evaluation manual and the standardized training manual for operating room nurses was formulated as per the actual situation in the operating room. Chen et al. [3] proposed the use of the conceive‐design‐implement‐operate model for nurse training along with dynamic comprehensive evaluations. In that study, evaluation for nurses covered theoretical knowledge, teaching performance, and the completion of nursing projects (proportion: 4:3:3). The results showed that this evaluation method was conducive for improving the postcompetency of new nurses but had to be used in combination with a corresponding training program. Zhang [4] revealed that special training modes, including knowledge and theory of the operating room, professional skill training, and teacher training, could shorten the learning time for new operating room nurses and greatly improve the operating room's emergency handling capacity compared with the traditional mode. Gao [5] observed that refined teaching based on the objectives‐goals‐strategies‐measures model effectively increased new operating room nurses' professional ability and fostered the reform and optimization of nursing teaching and management in the operating room. Another study [6] indicated that the four‐component instructional design mode was worthy of clinical promotion as it could facilitate rapid and healthy growth among junior nurses. In 2016, Xu et al. [7] constructed an evaluation index system for the training effectiveness of junior operating room nurses' core competence based on the Kirkpatrick model. This system included reaction indices (training demand, training content, teaching conditions, and logistics management), learning indices (theoretical knowledge, specialized skills, personal ability, and standardized training), behavior indices (specialized nursing and professional attitude), and result indices (organization benefit, professional competence, and personal benefits). The weight of the 60 three‐level indices was set using the analytic hierarchy process (AHP). However, no application of this evaluation system appears to be available. Importantly, indices should be designed to conform to the principle of feasibility, and excessive indices may create inconvenience for users. Therefore, the rationality of this index system needs further verification.

### 1.2 Foreign evaluation index of standardized training for operating room nurses

Because of the different medical environments locally and internationally, foreign countries have a strict access system for operating room nurses and different countries have different training programs [8]. For example, Periop 101 is a core training course for new nurses formulated by the Association of Perioperative Registered Nurses, which comprises patient safety, skin assessment, and equipment use; nurses can receive online training and posttraining evaluation using this model. The United States developed the Certified Nurse Operating Room Certification Exam for operating room nurses. Japan has also established a ladder education system for the clinical nursing practice ability with different training priorities at different stages, which requires hospital nursing education committees to organize learning review meetings in the first month, 6 months, and 1 year after new nurses are recruited. However, many foreign countries lack specific evaluation systems for the standardized training of operating room nurses.

The above studies highlight that different countries have their own systems for training and evaluation, although research on operating room nurses remains relatively limited. In China, most evaluations of training effectiveness focus on theoretical and practical exams, and less attention is paid to the weight of the index, even when an evaluation strategy is developed. However, this kind of evaluation index can only evaluate the effectiveness of short‐term theoretical knowledge input and cannot effectively evaluate long‐term theoretical application and practice. Furthermore, it cannot reflect nurses' behavioral changes after training and their actual contribution to elevating organizational efficiency, thereby discouraging the continuous improvement of training quality. Against this backdrop, it has become important for managers to develop a highly feasible and well‐popularized standardized training evaluation system that is suitable for the development trend of the surgical nursing specialty in China.

### 1.3 Theoretical basis

**Figure 1:**
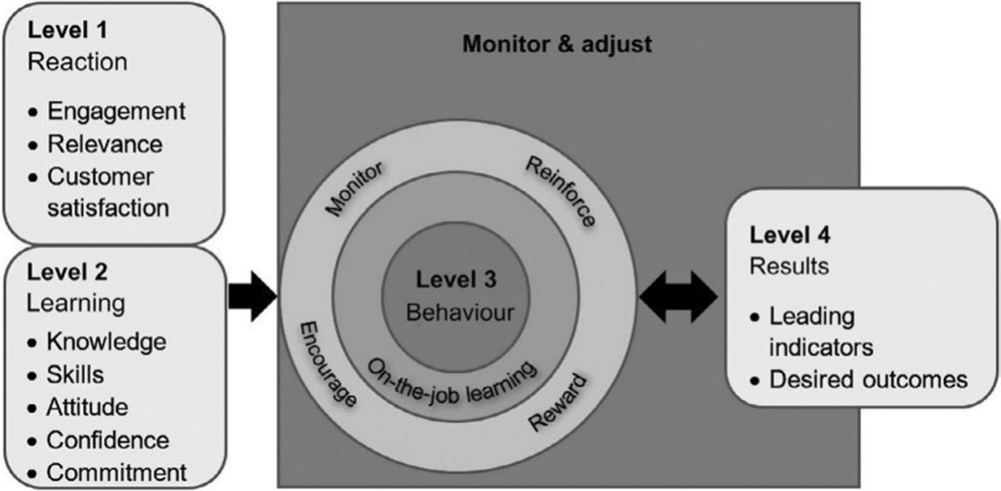
The new Kirkpatrick model [10].

Kirkpatrick's four‐level model is the earliest classic model in the evaluation field and is one of the most widely used and operational evaluation models [9]. This model is primarily used to evaluate trainees and offers an advantage over other training evaluation models in terms of time consumption. Originating from the concept of “experience‐gain‐application‐output”, the model evaluates training over four levels: reaction, learning, behavior, and result. This provides a systematic and comprehensive evaluation model with a clear hierarchy. In detail, the model evaluates: trainees' satisfaction with the training (reaction level); how much knowledge and skills trainees should master (learning level); the degree to which trainees apply their knowledge and skills in clinical work (behavior level); and the extent to which the training and corresponding reinforcement measures achieve the desired operational results (result level). Kirkpatrick [10] published an article commemorating the 50th anniversary of the Kirkpatrick four‐level model. This article aimed to improve training effectiveness by modifying the model and proposed several recommendations for training managers, including: training should start with results (business needs); training leaders should form a partnership with managers; and strengthening the transformation and consolidation of results after training is crucial for improving training effectiveness (Figure 1).

### 1.4 Aims

The principal purpose of this mixed‐method study is to develop and validate a system to evaluate training effectiveness for new nurses in the operating room in China based on the Kirkpatrick model.

### 1.5 Research questions

Our main research question is how to evaluate the effectiveness of standardized training for operating room nurses. Based on the operational levels of the Kirkpatrick model, specific research questions are as follows.

a. What content and form of training do new nurses want to receive (reaction)?
b. What theoretical knowledge and operational skills do operating room nurses acquire after standardized training from nursing managers' perspective (learning)?
c. From what aspects should we evaluate the impact of standardized training on the new nurses' clinical performance (i.e., nurses' knowledge, skills, and attitudes) (behavior)?
d. How can the impact of standardized training on the personal and organizational environments of new nurses be evaluated (results)?
e. Which indices should be included in the Structured Training Effectiveness Evaluation (STEE) tool and how should the weight of each index be determined?
f. Will the evaluation system increase the effectiveness of standardized training?

## 2 METHODS

**Figure 2:**
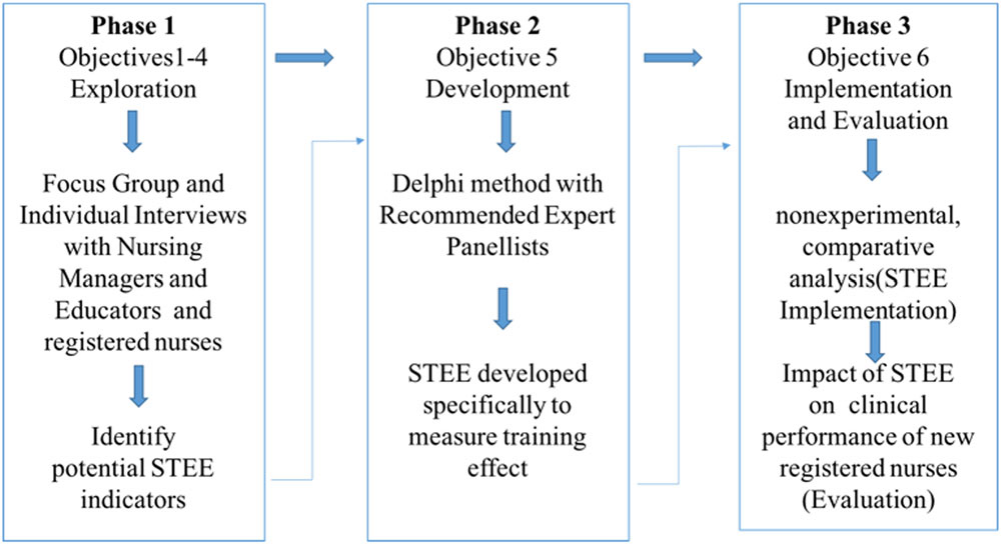
Study visual diagram. STEE, Structured Training Effectiveness Evaluation.

Approval for this protocol will be sought from the Ethics Committee of the People's Hospital of Peking University. The study will follow a sequential multiphase mixed‐method design, which will allow us to draw on the strengths from each approach while offsetting the limitations with the use of both quantitative and qualitative approaches. The research will be conducted in three phases (Figure 2).

Phase 1: The researchers will analyze the characteristics of an effective training program to identify a set of potential indices based on four levels (objectives 1–4).

Phase 2: Based on these potential indices, the STEE tool will be developed and refined using the Delphi method with a panel of experts.

Phase 3: The STEE tool will be implemented and evaluated through an exploratory, nonexperimental, and comparative analysis.

### 2.1 Phase 1: Qualitative study

This phase will involve a qualitative study to identify potential indices for inclusion in the STEE. In this phase, it is considered important to obtain the views of new nurses attending training from the outset. In addition, it is necessary to consult with nurse educators with experience in developing or implementing training programs (objectives 1–4).

#### 2.1.1 Samples

Several key stakeholders will be purposively selected for this study as they can provide information on particular objectives in the research questions (Table 1). New nurses, training educators, and surgeons will be selected as interviewees using a purposive sampling method.

**Table 1:** Inclusion/exclusion criteria for phase 1.

Objective 1: What content and form of training do new nurses want to receive (reaction)?	
Inclusion criteria:	Exclusion criteria:
(1) Subjects obtaining nurse qualification certificate; (2) subjects with work experience of ≥1 year and ≤2 years; (3) subjects with normal understanding and expression abilities; (4) subjects with bachelor's degree or above	(1) Subjects unwilling to participate in this study; (2) subjects participating in other related studies at the same time
Objective 2–4: What content and form of training do new nurses want to receive (reaction)?	
Inclusion criteria:	Exclusion criteria:
(1) Subjects in charge of nurse training for more than 5 years; (2) subjects with normal understanding and expression abilities; (3) subjects with bachelor's degree or above	(1) Subjects unwilling to participate in this study; (2) subjects continuing nursing at this hospital

#### 2.1.2 Sample size

Individual interviews will be performed with new nurses until data saturation is reached, and objective 1 will be accomplished as per the recommendation in a previous study [11]. The sample size will be determined by the principle of information saturation. As recommended by Harris et al. [12], three to four focus group interviews will be conducted, each with six to eight participants of similar professional status, to achieve objectives 2–4.

#### 2.1.3 Sampling

Operating room nursing educators will be selected using convenience sampling from 14 operating room nurse specialist training bases in Beijing that are accredited by the Chinese Nursing Association. Operating room nurses from our hospital will be selected by convenience sampling.

#### 2.1.4 Interview outline

The interview outline for nursing educators will include the following questions: (1) What elements do you think a high‐quality orientation for new operating room nurses should have? (2) What are the core theories and basic and specialized knowledge that new nurses should master after training and how can this aspect be evaluated? (3) What skills and nursing abilities do you believe new nurses should master after training and how can this aspect be evaluated? (4) What benefits do you think successful training can provide to nurses and departments?

The interview outline for clinical nurses will include the following key question: What do you think good, standardized training should look like (it can be guided by courses, trainers, training methods, and assessment methods)?

#### 2.1.5 Data collection

Participants will be invited to attend both a focus group and an individual interview. Participants will be contacted before the interview to explain the purpose of the interview and schedule an appropriate time and place. The interviews will be conducted in accordance with the interview outline and recorded with the interviewees' consent. Topic guides for both individual and focus group interviews will be piloted before the study starts. Focus group and semistructured individual interviews will be audio‐recorded and transcribed verbatim.

#### 2.1.6 Data analysis

Data will be analyzed using the NVivo qualitative analysis software and thematic analysis. Major coding will be performed based on the interview outline (Supporting Information S1: File 1). Any new codes that appear in an interview will be added and the name of each code will be determined. A description of the interview content will be added for each code. Potential process indices will be identified with analyzed data obtained from diverse key stakeholders combined with relevant literature [13, 14, 15, 16].

### 2.2 Phase 2: Delphi method

#### 2.2.1 Construction of evaluation indices and consultation questionnaire

Based on the expert selection criteria, consulting experts will be determined and consulted by letter. The results of each consultation round will be statistically analyzed and the opinions of experts will be fed back to these experts for reference before the next consultation round. This process will aim to achieve consistent opinions and the evaluation indices for standardized training effectiveness for operating room nurses will be obtained.

The expert questionnaire will be designed based on a literature review and phase 1 results, and include: (1) the questionnaire instructions, which will explain the background, objectives, and significance of the research; (2) the basic information about experts, including their department, age, post, title, research direction, working years, and supervision of postgraduate/doctoral candidates or not; (3) an expert self‐evaluation form, including completion requirements, the degree of experts' familiarity with the questionnaire content, and the evaluation basis of the self‐evaluation form; (4) an expert opinion and suggestion table, including the evaluation index system for nurse training effectiveness comprising a three‐level structure, where different index items will be evaluated by experts according to their importance. Using a 5‐point Likert scale, the “importance” of items will be graded as 5 (very important), 4 (important), 3 (generally important), 2 (not very important), and 1 (not important). Furthermore, a column for modification comments will be included, in which experts can propose suggestions for modification, addition, or deletion of items.

#### 2.2.2 Identification of experts

##### 2.2.2.1 Inclusion criteria

The inclusion criteria for the experts are: (1) experts with an intermediate title or above; (2) experts in charge of the training of operating room nurses in third‐level grade A hospitals; and (3) experts with at least 10 years of clinical, management, or education experience.

##### 2.2.2.2 Number of experts

Based on the Delphi method, there should be 15–50 experts. Therefore, our study will include 30 experts from the clinical, management, and education fields in Beijing (10 experts per field). Before consultation, the background and methods of our study will be explained to the experts to obtain their support and cooperation.

#### 2.2.3 Implementation

The questionnaire will be distributed by email, WeChat, or face‐to‐face and collected within 14 days. The researchers will then collate, analyze, discuss, and modify the experts' opinions and suggestions using the feedback from each consultation round to form the questionnaire for the next round and will feed back the results of the previous consultation round to each expert for reference in the next round of evaluation.

#### 2.2.4 Entry screening criteria

In this study, the items will be included if they have a mean importance value of >4 scores and a variation coefficient of <0.25 (the maximum mean value of importance will be 5). To ensure the rationality and clinical practicability of items, the items will be strictly screened based on the criteria and determined through collective discussion by the research group combined with consideration of clinical practice and expert opinions [17].

#### 2.2.5 Weight of the items

In the third round, experts will be asked to determine the importance of the score results of each index using AHP and calculate the importance of each item and the differences between the importance of each degree. The hierarchical structure model will be established with the AHP technique and divided into three layers.

#### 2.2.6 Data analysis

Data entry and statistical analysis will be performed with Excel and the AHP5.0 software package. The Delphi method will reflect the authority and reliability of the results through the positive coefficient and authority degree of experts and the concentration and coordination degrees of expert opinions. The general situation of experts will be expressed with descriptive statistics (percentages and frequencies). The positive coefficient of experts will be summarized as the effective recovery rate of the questionnaire. The experts' degree of authority will be represented by the authority coefficient. The concentration degree of expert opinions will be presented by the mean value of index importance assignments and full score ratios. Finally, the coordination degree of expert opinions will be shown by the expert coordination coefficient (Kendall's *W*).

### 2.3 Phase 3: Quantitative study

This phase (objective 6) will involve an exploratory, nonexperimental, and comparative analysis with the newly developed STEE tool as the measurement instrument. We propose that the STEE tool will have four components: (1) reaction level (nurse satisfaction); (2) learning level (theoretical knowledge and operational skills); (3) behavior level (clinical competence and professional attitude); and (4) result level (personal growth and department development). We will compare new nurses enrolled in August 2017 and August 2018 who did not use the STEE tool with new nurses enrolled in August 2019 and August 2020 who used the STEE tool. First, we will establish a standardized training evaluation team, including standardized training implementers, nursing managers, and surgeons.

#### 2.3.1 Samples and setting

The quantitative study participants will include all newly graduated registered nurses from 2017 to 2020 in the People's Hospital of Peking University, which is a third‐level grade A comprehensive hospital committed to delivering state‐of‐the‐art clinical care, innovative scientific research, and rigorous medical education. Participants meeting four criteria will be included: (1) new operating room nurses; (2) nurses who pass the nurse qualification exam; (3) nurses with no working experience in other hospitals; and (4) nurses who volunteer to participate in this study.

#### 2.3.2 Instruments

Based on the results of the first and second study phases, the corresponding evaluation tools will be developed with the evaluation indices through a literature review, and the indices will be classified into subjective and objective indices. Objective indices, including theoretical examination results and the incidence of adverse events, should be evaluated objectively by evaluation teachers or collected from department information. Subjective indices include nurses' cooperation ability and relevant information will be collected via a questionnaire survey (both self‐evaluation and evaluation by others). The questionnaires will mainly cover general information and training effectiveness evaluation. (1) The general information questionnaire will be designed by the researchers based on a literature review to understand participants' basic situation. The self‐evaluation questionnaire will include age, gender, education background, intern status, and marital status, and the questionnaire for evaluation by others will comprise age, gender, title, post, and department. (2) The standardized training nurse evaluation questionnaire will be transformed from the third‐level index elements in the standardized training effectiveness evaluation system preliminarily designed for new nurses. The total score will be calculated using a 5‐point Likert scale (“*completely consistent*” = 5 and “*completely inconsistent*” = 1) based on the weight of each item. A higher score will indicate more effective training.

#### 2.3.3 Data collection

The QR code for the questionnaire will be formed using the WJX platform, and the completion authority will be set to prevent item omissions. Participants will scan the QR code and complete the questionnaire on their own. The evaluation contents and participants will differ at different levels: (1) *reaction level*: the questionnaire will be completed by nurses after 1 and 2 years of standardized training; (2) *learning level*: the questionnaire will be completed by nurses and evaluation group members after 1 and 2 years of standardized training; and (3) *behavior level*: the questionnaire will be completed by evaluation group members and surgeons after 1 and 2 years of training.

#### 2.3.4 Data analysis

The database will be established using Epi‐Data 3.1 statistical software. All questionnaire data will be entered synchronously after double‐checking. In addition, 5% of the questionnaires will be randomly checked to ensure correct data entry. Next, the data will be imported into SPSS version 23 to screen the whole database and conduct statistical analyses:

a. The general information of the research participants and the scores for each item will be statistically analyzed. Measurement data with normal distribution will be expressed as mean ± standard deviation, and nonnormally distributed data will be summarized as median and quartile. Enumeration data will be described as rates and percentages. Two‐sided tests will be used and the test level (*α*) will be set at 0.05.
b. Inferential analyses will be performed with a series of Pearson's *χ* ^2^ tests to determine the relationships between the STEE tool and the results to clarify whether the variation is caused by chance or the variable being tested.

## 3 DISCUSSION

This study will clarify how to evaluate the effectiveness of standardized training for new nurses. It will gather the perspectives of key health professionals in China who are familiar with the development and implementation of nursing training programs but also information on how to develop a valid and reliable STEE tool using the Delphi method. This mixed‐method study will use both qualitative and quantitative research methods to comprehensively understand the impact of the STEE tool on the effectiveness of standardized training for new nurses.

In this study, each method is selected because of its ability to address a particular objective and aspect of the study. In contrast, using only one method for the study would significantly limit our ability to comprehensively address all of the study objectives.

Based on the Kirkpatrick model, this study will construct a scientific and feasible standardized training effectiveness evaluation system for new nurses. This study will provide guidance for the further improvement of standardized training methods in the operating room, method references for related further research, a theoretical basis for improving the evaluation system, and a reference for managers to perform training effectiveness evaluation. Overall, this will increase training effectiveness and ensure the quality of nursing services.

## 4 LIMITATIONS

The following limitations may exist in this study. In the qualitative phase, nurses from one hospital may affect the reaction level. We will attempt to fully understand their needs using group interviews. In addition, the sample size of this planned study may restrict the generalizability of the expected results. Finally, because there is no stage evaluation before implementation of the STEE in the last phase, comparisons of the effect can only be conducted after the end of the evaluation.

## AUTHOR CONTRIBUTIONS

Yanshu Wei completed the study design. Xiaoli Liu, Xiaozhou Wu, and Jin Pei completed the data analysis. Xiaoli Liu completed manuscript editing. All authors read and approved the final manuscript.

## CONFLICT OF INTEREST

The authors declare no conflict of interest.

## ETHICS STATEMENT

This study will be authorized by the Regional Ethics Committee of Peking University People's Hospital. The study conforms to the Declaration of Helsinki. The nurses, nursing educators, and experts will be asked for voluntary participation. Written informed consent will be obtained for interviews/focus groups and the Delphi method.

## INFORMED CONSENT

None.

## Supporting information

Supporting information.

## Data Availability

Data sharing is not applicable to this article as no data sets were generated or analyzed during the present study.
