# Supplementary material for: Construction and application of standardized training effect evaluation system for new nurses in operating room
Source: Health Care Sci. 2023 Dec 10;2(6):392–9. doi: 10.1002/hcs2.75 (PMC11080677; doi:10.1002/hcs2.75)
Supplement: Supplementary file 1 — Supporting information. [file HCS2-2-392-s001.docx]

Supplementary File 1 The interview outline proposed based on the purpose of the study:

| 1 | What elements do you think should be present in a high-quality orientation for new operating room nurses? |
| --- | --- |
| 2 | What core theories and basic and specialized knowledge related to the job do you think new nurses should have at the end of standardized training? |
| 3 | What skills and nursing capabilities do you think new nurses should have at the end of standardized training? |
| 4 | What benefits do you think successful training can bring to nurses and the department? |
| 5 | From what aspects do you think the above capabilities can be evaluated? |
